# Single-session measures of quadriceps neuromuscular function are reliable in healthy females and unaffected by age

**DOI:** 10.1007/s00421-023-05395-x

**Published:** 2024-01-08

**Authors:** Steven J. O’Bryan, Danielle Hiam, Séverine Lamon

**Affiliations:** 1 Institute for Physical Activity and Nutrition (IPAN), School of Exercise and Nutrition Sciences, Deakin University, 221 Burwood Highway, Burwood, Geelong, VIC 3125 Australia; 2 Institute for Health and Sport, Victoria University, Melbourne, Australia

**Keywords:** Electrical stimulation, Strength, Validity, Skeletal muscle, Motor unit, Aged

## Abstract

**Purpose:**

This study aimed to determine the inter-session reliability of quadriceps neuromuscular function measurements in healthy young and older females.

**Methods:**

Twenty-six females aged 19–74 years completed two identical experimental sessions on different days. Quadriceps neuromuscular function measurements included isometric maximal voluntary force, high- and low-frequency twitch force, voluntary and evoked (H-reflex, M-wave) electromyography (EMG), and estimated maximal torque, velocity and power derived from torque–velocity relationships. Intra-class correlation coefficients (ICCs), coefficients of variation (CoV) and Bland–Altman plots assessed inter-session reliability. The effect of age on reliability was assessed by linear regression.

**Results:**

Excellent reliability (ICC > 0.8) was shown for all voluntary and evoked mechanical outcomes. *Vastus lateralis* EMG outcomes showed excellent reliability (ICC > 0.8) with CoVs < 12%, which were better than those of *vastus medialis* and *rectus femoris*. Age was not associated with reliability for 27/28 outcomes (*P* > 0.05).

**Conclusion:**

Excellent reliability of voluntary and evoked force and *vastus lateralis* EMG outcomes measured in healthy females can be attained in one experimental session, irrespective of age. Female neuromuscular function can be accurately assessed across the lifespan with minimal inconvenience, increasing feasibility for future research. The random error should however be considered when quantifying age-related differences in neuromuscular function.

## Introduction

The degenerative effects of the ageing neuromuscular system and the effectiveness of interventional strategies aimed at improving motor function are grounded in cross-sectional or longitudinal observations. For example, some studies demonstrate an annual reduction in strength of ~ 1–4% past the age of 65 years (Frontera et al. 2000), whereas others show a ~ 15–40% reduction in muscle shortening velocity and a ~ 10–30% reduction in power in older compared to younger adults (McNeil et al. 2007). Yet, no studies have concomitantly evaluated the inter-session reliability of a wide range of valuable measures used to assess neuromuscular function at different stages of the lifespan, despite well-known age-related variability in neuromuscular structure arising from biological (e.g. genetic, inflammatory) or lifestyle (e.g. physical activity, nutritional) factors (O'Bryan and Hiam 2022). Moreover, although more research specific to female neurophysiology is emerging, test–retest reliability of neuromuscular function has not been established in females despite sex-specific differences in steroidal hormones which fluctuate during the menstrual cycle/menopause and can excite (e.g. oestrogen) or inhibit (e.g. progesterone) the central nervous system (Guo et al. 2022; Lulic-Kuryllo and Inglis 2022; Gómez-Cabello et al. 2014). Thus, quantifying the random error in neuromuscular function measurements obtained from females at different stages of the lifespan will allow to appropriately interpret repeated and cross-sectional observations and inform feasibility in future research.

Neuromuscular function is characterized by the translation and transmission of synaptic inputs received by motoneurons into the generation of external forces necessary for human movement. At a general level, neuromuscular function may assessed by quantifying maximal force/torque generated during isometric (Gómez-Cabello et al. 2014), isokinetic (Pincivero et al. 1997) or isoinertial (do Nascimento et al. 2013) voluntary contractions. In each instance, reported inter-session reliability was high in younger and older males and females (intra-class correlation coefficient > 0.88) (Symons et al. 2004; Holsgaard Larsen et al. 2007; Place et al. 2007; Behrens et al. 2017; Zech et al. 2008; Jenkins et al. 2014; Buckthorpe et al. 2012; Behm et al. 1996; Blacker et al. 2013), although isoinertial contractions may be less reliable (Nuzzo et al. 2019). Despite the high reliability of such measures of muscle strength, these tests are limited in their capacity to concurrently evaluate maximal shortening velocity and power production, which is arguably closer related to functional activities than maximal strength (Byrne et al. 2016; Samozino et al. 2012). Thus, others have evaluated age-related deficits in neuromuscular function by modelling torque–velocity (T–V) and power–velocity (P–V) relationships derived from a series of maximal dynamic contractions (single or multi-joint) performed at different isoinertial loads or isokinetic velocities (Alcazar et al. 2017; Callahan and Kent-Braun 2011; Clémençon et al. 2008). However, the reliability of T–V and P–V outcomes from a large functional muscle such as the quadriceps has not been established. Quadriceps function is strongly related to lower-limb mobility and power production across the lifespan (Callahan and Kent-Braun 2011; Clémençon et al. 2008). Coupling T–V and P–V relationships of quadriceps with techniques such as electrical nerve stimulation and surface electromyography (EMG) provides deeper knowledge regarding the neurophysiological properties that govern maximal force, velocity and power, beyond what can be obtained from maximal voluntary contractions alone (Millet et al. 2011).

A superimposed stimulus applied to the nerve during maximal voluntary contraction can evaluate the capacity of cortical and spinal motoneurons to activate muscle fibres and achieve maximal voluntary force (i.e. voluntary activation) (Taylor 2009), whereas high- and low-frequency stimulation at rest can evaluate mechanisms which influence neuromuscular transmission and excitation–contraction coupling (Hunter et al. 2016; Millet et al. 2011). Moreover, EMG recordings of compound muscle action potentials (i.e. M-wave) and Ia afferent reflexive responses (i.e. H-reflex) provide details on the capacity and velocity of action potential propagation (Millet et al. 2011) and muscle spindle excitation of alpha motoneurons (Theodosiadou et al. 2023). Despite the considerable value of electrical stimulation in assessing neuromuscular function in older populations (Hunter et al. 1998, 2016), the inter-session reliability of its outcomes has primarily been investigated in younger males (Place et al. 2007; Behrens et al. 2017; Zech et al. 2008; Jenkins et al. 2014; Buckthorpe et al. 2012; Behm et al. 1996; Blacker et al. 2013) and once in older males and females with knee osteoarthritis (Staehli et al. 2010). Age and sex both have influence on the neurophysiological and morphological features of the superficial quadriceps (Mizuno et al. 2021; Guo et al. 2022), indicating that inter-muscular differences may exist in the inter-session reliability of EMG outcomes and warranting more specific investigations.

The aim of this study was to determine the test–retest reliability of quadriceps neuromuscular function in healthy females between 18 and 80 years of age. Maximal isometric contractions, low- and high-frequency doublets, force–velocity and power–velocity relationships during dynamic contractions and voluntary and evoked surface electromyography responses (root mean square [RMS EMG], M-wave and H-reflex) were assessed during two identical experimental sessions. In addition, inter-muscular differences in the reliability of EMG outcomes between the superficial quadriceps were evaluated.

## Method

### Participants

A required sample size of 23 was calculated with an expected ICC of 0.9, a minimum ICC of 0.7 and a statistical power of 80% (Holsgaard Larsen et al. 2007). An additional 10% were recruited to account for participant dropout or missing data points. Twenty-six healthy females aged 19 – 74 years (mean ± standard deviation: age = 43 ± 18 years, height = 164 ± 5.7 cm, weight = 63.5 ± 10.8 kg and BMI = 23.5 ± 3.9 kg.m^2^) volunteered to participate in this study. Two participants did not engage in physical exercise, eight performed light-intensity exercises (e.g. Pilates, golf and walking), nine were moderately trained (e.g. resistance exercise and jogging) and seven regularly performed vigorous exercise (e.g. competitive hockey, high-intensity running and cross-training). Seventeen participants were pre-menopausal, with ten of those with an implanted intrauterine device or taking oral contraceptives. The remaining ten participants were post-menopausal, with one participant on hormone replacement therapy. Prior to participation, a medical history and risk-factor assessment questionnaire was completed to confirm eligibility. The exclusion criteria included pregnancy, cancer, implanted medical devices, BMI > 35 kg.m^2^ or any diseases of the central nervous system, musculoskeletal system, cardiorespiratory system or metabolic system. A written informed consent was obtained from each participant prior to the commencement of the study. All testing procedures were approved by the Deakin University Human Research Ethics Committee (DUHREC 2021-307).

### Experimental protocol

Participants visited the laboratory on two separate occasions at the same time of day, with a duration of 9 ± 7 (mean ± standard deviation) days separating each visit. The participants were instructed to avoid strenuous physical activity in the 48 h preceding each visit and to avoid caffeine on the day of testing.

All experimental testing was conducted on an isokinetic dynamometer (Universal Pro Single Chair model 850-230, Biodex Medical Systems, United States). The participants sat upright in the dynamometer chair with straps across the thorax and pelvis, with hip angle set at 85° flexion. The axis of rotation of the dynamometer was aligned with the axis of rotation of the dominant knee (estimated by palpation of the lateral femoral epicondyle), with the distal aspect of the dynamometer attachment fixed to the leg via a Velcro strap ~ 2cm proximal to the lateral malleoli of the fibula. The participants were instructed to cross their arms across their chest during all testing.

**Fig. 1:**
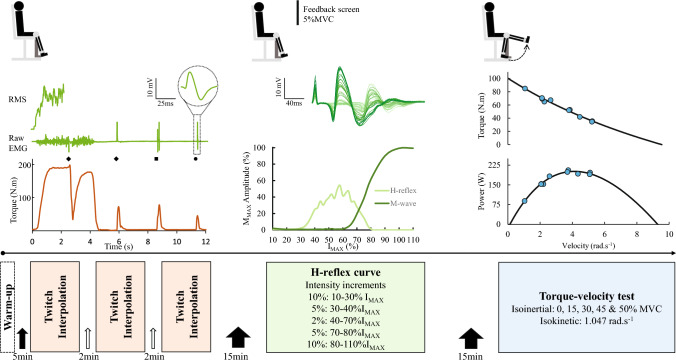
Timeline of the experimental protocol. Following the generalized warm-up, the twitch interpolation procedure was administered three times with 2 min rest. Torque and raw EMG of the superficial quadriceps (*vastus lateralis* example) were synchronously recorded, with RMS applied to raw EMG during MVC. High-frequency 100 Hz doublets (diamonds), low-frequency 10 Hz doublet (square) and 1 Hz single pulse (circle) were applied to the femoral nerve. M-wave amplitude and duration were examined from the single-pulse EMG response (inset). Following 15-min rest, H-reflexes were elicited in the quadriceps by constructing a stimulus response curve during 5% MVC background contraction. Fifty stimulations were delivered at progressively increasing intensity, with 10-s separating each contraction. An example raw trace of all overlaid responses is shown, with lighter lines representing responses at lower stimulation intensities. The peak-to-peak M-wave amplitude and H-reflex were plotted to construct a stimulus response curve. Following another 15-min rest period, torque–velocity and power–velocity relationships were constructed by plotting torque and power against velocity (blue dots) measured over a series of isoinertial and one isokinetic contractions

Following a standardized warm-up consisting of a series of incremental submaximal and maximal isometric knee extensions (one 4 s contraction at 20%, 40%, 60% and 80% perceived effort and up to three at 100% perceived effort), maximal voluntary and evoked mechanical and electromyography responses were measured in quadriceps with three experimental protocols separated by 15 min of rest (Fig. 1). First, the participants completed a ⁓4 s maximal isometric voluntary contraction (MVC) at 75° knee flexion with an electrically evoked doublet (100 Hz) applied to the femoral nerve at the plateau in voluntary force, followed ~ 2 s after by three resting evoked responses (100 Hz and 10 Hz doublet plus a 1 Hz single pulse, ~ 1.5 s apart) (i.e. twitch interpolation). This procedure was repeated three times with two minutes rest separating each set. Second, Hoffmann (H) reflexes were elicited in the quadriceps by applying a 1 Hz electrical stimulus to the femoral nerve at progressively increasing intensity during 50 brief (2–3 s) submaximal isometric contractions performed at 5% MVC (~ 10 s rest separated each contraction with inter-stimulus duration = 13.6 ± 0.9 s). The participants were asked to keep their head straight with eyes on the torque feedback screen, place their arms across the chest, avoid any unnecessary limb movements and remain quiet. Finally, torque–velocity and power–velocity relationships of the quadriceps were derived from maximal voluntary contractions performed at five different isotonic loads (~ 0% MVC, 15% MVC, 30% MVC, 45% MVC and 50% MVC) and one isokinetic speed (1.047 rad/s). The participants were instructed to extend their knee as hard and fast as possible from 110° to 10° knee flexion (0° = full extension), with two efforts performed for each load/speed (⁓3 s apart) and 3 min rest between different load/speed. Strong verbal encouragement was provided for all maximal efforts.

### Electrical stimulation

Electrical stimulation of the femoral nerve was induced with a constant-current electrical stimulator (model DS7AH, Digitimer, Welwyn Garden City, UK) and custom-built pulse frequency generator. Voltage was set to maximum (400 V) and pulse duration to 1 ms. A handheld ball-point cathode wrapped in gauze and soaked in saline (20 mm diameter) and a 90 mm × 50 mm self-adhesive rectangular anode placed midway between the superior aspect of the iliac crest and the greater trochanter of the femur were used to stimulate the femoral nerve. At the beginning of each session, the location of the femoral nerve in the femoral triangle was determined as the position where the quadriceps twitch force and M-wave responses were highest at a stimulation intensity of 50 mA. This site was marked with indelible ink to ensure the stimulation site remained optimal and consistent throughout the experiment. A stimulus response curve was constructed by measuring the peak torque and peak-to-peak M-wave amplitude elicited from stimulations delivered at 10 mA increments from 50 mA up to the intensity which elicited no further increase in the responses (I_MAX_). Stimulation intensity for maximal evoked responses was set to 130% I_MAX_ (140 ± 28 mA). For H-reflexes, the stimulation intensity began at 10% I_MAX_ and was increased after every second contraction (i.e. two stimulations at each intensity) by 10% between contractions 1 and 6, 5% between contractions 6 and 10, 2% between contractions 10 and 40, 5% between contractions 40 and 44 and 10% between contractions 44 and 50. The H-reflex protocol was slightly modified from previous protocols (Doguet and Jubeau 2014) by implementing relative increases in stimulation intensity and by asking participants to momentarily hold a 5% MVC background torque level whenever stimulations were applied. This approach avoided between-participant variations in the number of contractions/stimulations throughout the procedure. Moreover, piloting within a sample of our cohort demonstrated that background torque levels > 5% MVC led to fatigue development (via decrease in MVC) in some participants, which is known to impact the H-reflex response (Theodosiadou et al. 2023).

### Surface electromyography

Disposable pre-gelled Ag–AgCl surface electrodes (Blue sensor N, Ambu, Ballerup, Denmark) were used to record EMG signals unilaterally for *vastus lateralis* (VL), *vastus medialis* (VM) and *rectus femoris* (RF) in a bi-polar arrangement. A single 20-mm Ag–AgCl ground electrode (Conmed ClearTrace ECG sensor electrode, Utica, NY) was attached to the lateral malleoli of the opposite ankle. Bi-polar surface electrodes were positioned at an inter-electrode distance of 20 mm and aligned parallel to the muscle fibres in accordance with the recommendations of the SENIAM project (Hermens et al. 2000). Prior to the placement of electrodes, the skin was prepared by shaving, abrading and cleaning with an alcohol swab. The EMG electrodes were secured with an adhesive tape to ensure good contact with the skin and to reduce movement artefact. Indelible ink was used to mark electrode location at the end of the first session, and the participants were asked to avoid removing the ink before the second session, ensuring that the electrode location was consistent between sessions.

### Data acquisition and analysis

All torque (N.m), angle (rad), velocity (rad.s^−1^), EMG (mV) and stimulation pulse analogue data were synchronously acquired at 4000 Hz and digitally converted with a Powerlab 8/30 (ADinstruments, Australia) in conjunction with a PC running Labchart software (version 8.1.24, ADinstruments, Australia). Raw EMG signals were amplified (gain: 1000, BioAmp ADinstruments, Australia) and processed with a bandpass filter (fourth order Butterworth 20–500 Hz) and 50 ms root mean square (RMS EMG) for data recorded during MVC. All offline processing was completed using Spike2 software (version 7.13, Cambridge Electronic Design, Cambridge, UK).

Gravity correction of the recorded isometric torque data was applied by adding the torque generated by the passive weight of the leg and dynamometer attachment at 75° knee flexion. During MVC, the average torque (N.m) and RMS EMG (mV) were calculated over a 500 ms time window preceding superimposed stimulation, and superimposed twitch torque (SIT) was calculated as the change in torque within an ~ 120 ms time window following stimulation. From the potentiated resting twitches at 100 Hz, 10 Hz and 1 Hz, peak torque (PT), maximal rate of torque development (RTD – maximum of the torque–time slope following 100 Hz stimulation (1 ms time constant)) and the ratio between PT_100Hz_ and PT_10 Hz_ (PT_100:10_) were calculated. The equation $1-\left(\frac{SIT}{Pt100HZ}\times 100\right)$ was used to calculate voluntary activation (%) (Taylor 2009). The average value from all the three assessments was used for analysis unless MVC force was < 90% of the highest value recorded in a particular session (in which case it was discarded).

From the maximal M-wave responses obtained from the raw EMG during 1 Hz resting twitches, peak-to-peak amplitude (M_MAX_) and peak-to-peak duration (M_DUR_) were calculated for all quadriceps muscles. RMS EMG during MVC was normalized to M_MAX_ (RMS.M_MAX_ %) to remove the contribution of peripheral factors on the RMS EMG signal and to permit better representation of overall neural drive (Millet et al. 2011; Farina et al. 2004). From the H-reflex procedure, peak-to-peak amplitude of the H-reflex and M-wave was calculated from each stimulation given to construct a stimulus response curve (Fig. 1). H_MAX_ was determined as the highest peak-to-peak amplitude relative to maximal M-wave amplitude (H_MAX_.M_MAX_) (Doguet and Jubeau 2014).

Concentric torque data were gravity offset by multiplying the passive torque generated by the weight of the leg at 10° flexion (0 = full extension) by the cosine of the recorded knee angle. An additional power channel (W) was calculated by multiplying torque (N.m) by velocity (rad.s^−1^). For all maximal efforts, average torque, velocity and power were extracted from torque onset (3 × standard deviations above baseline values) to knee angle of 30° flexion. This angle was chosen to avoid increased resistance generated by the braking torque (i.e. cushioning) of the dynamometer towards full knee extension during high-velocity contractions. The average value was chosen over the peak due to better reliability in the outcomes and closer associations with physical function (Alcazar et al. 2017). Torque–velocity (T–V) relationships were modelled by re-writing the original hyperbolic equation developed by Hill (1938) (non-linear least squares method):

1 $$T= \frac{c}{\left(V+b\right)}-a,$$

where T is the torque produced at a given velocity V, and a, b and c are constants. From the T–V relationship, the following variables were extracted: 1) maximal torque (T0) at the y-axis intercept when V = 0, 2) maximal velocity (V0) at the x-axis intercept calculated as $T0 \times \left(\frac{b}{a}\right)$, 3) optimal torque (T_OPT_) at maximal power calculated as ${\left({a}^{2}+\left(a\times T0\right)\right)}^{0.5}-a$, and 4) optimal velocity (V_OPT_) at maximal power calculated as $\frac{\left(\left(-a\times b\right)-\left(b\times Topt\right)+c\right)}{a+Topt}$ (1) (Hauraix et al. 2017). Power–velocity relationships were also modelled using the following equation:

2 $$P=bT\left(\left(\frac{c+a}{T+a}\right)-1\right),$$

where P is power produced at a given torque and velocity T, and a, b and c are constants. Maximal power (P_MAX_) occurred at the apex of the power–velocity relationship and was calculated as $\left(b\times Topt\right)\times \left(\left(\frac{c+a}{Topt+a}\right)-1\right)$ (Tihanyi et al. 1982). The single isokinetic effort at 1.047 rad/s (i.e. 60 deg/s) was included in the modelling procedure to increase the validity of torque estimates at low-contraction velocities. This could not be achieved with an isoinertial load beyond 50% MVC, as there was large inter-individual variability in the capacity for participants to achieve the required range of motion due to the limiting effects of the skeletal muscle length–tension relationship. Both efforts completed at each load/velocity were included in modelling procedures unless they deviated by > 5%, in which case, the highest value was included.

### Statistics

All statistical analyses were performed using R Software (v4.2.2; R Core Team 2022). Normal distribution of the original data and delta between sessions was assessed with histograms, Q–Q plots and Shapiro–Wilk test, with natural log transformations applied if data displayed a non-normal distribution. Homoscedasticity of all data was confirmed with the Breusch–Pagan test. Generalized linear mixed models with fixed effect of session (one or two) and random effect of participants assessed differences in the outcomes between sessions. An additional fixed effect of muscle was included for EMG outcomes to determine differences in the delta between sessions between *vastus lateralis, vastus medialis* and *rectus femoris* muscles (i.e. session × muscle interaction). The relationship between delta in the outcomes between sessions with age of the participants was assessed using linear regression. Significance was set at *P* < 0.05 for all statistical tests.

Relative reliability (i.e. participant position within the sample) between sessions one and two was assessed by the intra-class correlation coefficient (ICC) with a two-way random-effects model for single-measure reliability. ICC values between 0.8 and 1 were considered ‘excellent’, 0.6 and 0.8 as ‘good’ and < 0.6 as ‘poor’ (Bartko 1966). Absolute reliability (i.e. variability in participant scores) between sessions one and two was assessed by calculating the typical error, coefficient of variation and the mean difference with 95% limits of agreement. Typical error (TE) in the original unit of measurement was calculated by dividing the SD of the absolute difference between sessions one and two by $\sqrt{2}$, and the coefficient of variation (CoV) in percent was calculated with the formula $\left(SD/Mean\right)\times 100$. Bland–Altman plots were constructed to determine the systematic error (i.e. bias towards higher or lower outcomes during sessions one or two) and random error (i.e. inherent biological or mechanical variations), by plotting the individual participant difference in the outcome between sessions one and two against the mean and the 95% lower and upper limits of agreement (LOA), respectively (Atkinson and Nevill 1998). If log transformation of original data was required, the antilog of the mean difference and LOA was used to transform log values to a percentage difference (Atkinson and Nevill 1998). LOA ratios (%) were calculated to allow direct comparison with previous results when necessary (Atkinson and Nevill 1998).

## Results

### Isometric and evoked forces

**Fig. 2:**
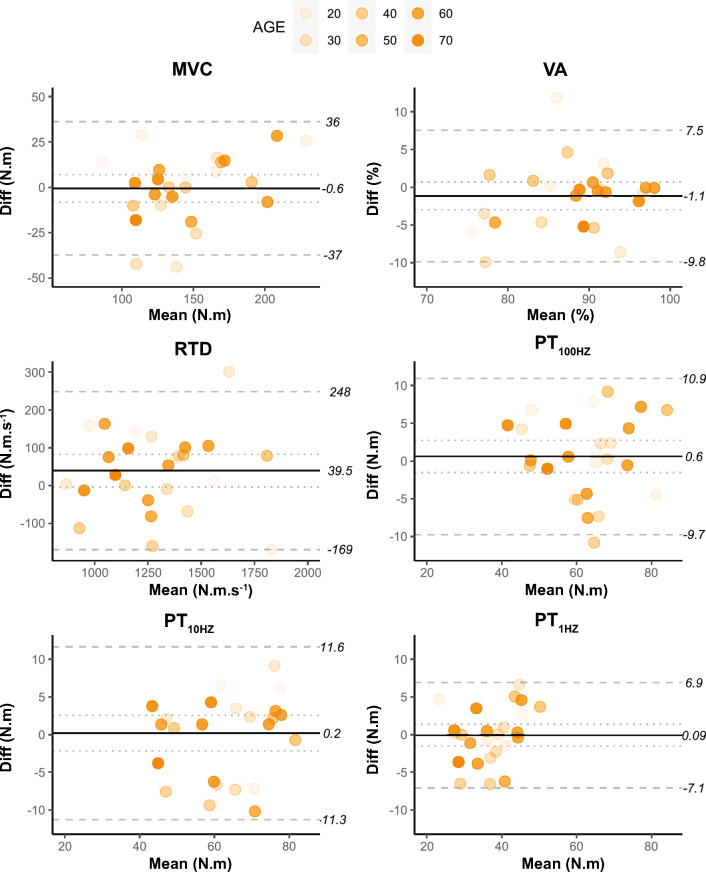
Bland–Altman plots with 95% level of agreement for voluntary and evoked forces. The mean value for sessions one and two is plotted on the x-axis, whereas the session one minus session two difference (Diff) is plotted on the y-axis. The solid line represents the grand mean difference for all participants, and the dotted line shows the 95% confidence interval. The dashed lines represent the lower and upper limits of agreement in original unit of measurement. Shading of the individual measures increases as a function of age. Sample size (n) for each variable: MVC (26), VA (25), RTD (26), PT_100HZ_ (26), PT_10HZ_ (26), PT_1HZ_ (26)

Isometric voluntary and evoked force measurements were similar between the sessions (all *P* > 0.05). All outcomes displayed ICCs > 0.8 (Table 1). CoVs were 2.6% for VA and < 5.5% for peak twitch forces and RTD. No notable systematic bias was observed for any of the voluntary or evoked force outcomes (Fig. 2).

**Table 1:** Mean ± standard deviation, inter-session reliability and regression (session delta vs. age) for all outcome measures

	Session #1	Session #2	Inter-session reliability			Age effect
	Mean (SD)	Mean (SD)	TE	CV (%)	ICC (lower, upper 95% CI)	*R*^2^
MVC (N.m)	144.85 (39.05)	146.77 (32.74)	13.26	7.24	0.869 (0.728, 0.939)	0.08
SIT (N.m)	7.92 (4.83)	7.36 (4.12)	1.78	18.35	0.842 (0.677, 0.927)	0.09
VA (%)	87.44 (7.49)	88.48 (6.48)	3.13	2.60	–	0.14
PT_100Hz_ (N.m)	63.04 (11.27)	62.53 (11.06)	3.72	4.79	0.893 (0.777, 0.951)	< 0.00
PT_10Hz_ (N.m)	62.3 (12.69)	62.5 (12.49)	4.13	5.62	0.897 (0.783, 0.952)	0.06
PT_1Hz_ (N.m)	37.11 (7.67)	37.27 (6.54)	2.51	5.26	0.881 (0.752, 0.945)	< 0.00
PT_100Hz:10 Hz_ Ratio	1.02 (0.11)	1.01 (0.09)	0.04	3.12	0.847 (0.69, 0.928)	0.02
RTD (N.m.s^−1^)	1311.5 (2260.5)	1279.3 (255.5)	75.40	4.92	0.908 (0.799, 0.958)	0.04
*Vastus lateralis*						
RMS EMG (mV)	0.217 (0.119)	0.220 (0.108)	0.02	9.22	0.95 (0.89, 0.978)	0.01
RMS.M_MAX_ (%)	5.08 (2.61)	5.24 (2.60)	2.09	10.97	0.846 (0.667, 0.933)	0.06
H_MAX_.M_MAX_ (%)	23.22 (14.14)	23.02 (16.50)	3.69	11.83	0.966 (0.914, 0.987)	0.13
M_MAX_ (mV)	4.52 (2.34)	4.62 (2.23)	0.52	8.34	0.945 (0.872, 0.977)	0.02
M_DUR_ (ms)	9.87 (2.9)	10.03 (2.75)	1.0	4.09	0.963 (0.915, 0.985)	< 0.00
*Vastus medialis*						
RMS EMG (mV)	0.35 (0.22)	0.36 (0.20)	0.03	7.89	0.966 (0.927, 0.985)	0.13
RMS.M_MAX_ (%)	3.45 (1.43)	3.7 (1.20)	0.65	14.39	0.758 (0.528, 0.885)	< 0.00
H_MAX_.M_MAX_ (%)	19.51 (13.49)	22.59 (13.71)	4.88	22.39	0.857 (0.656, 0.945)	0.42*
M_MAX_ (mV)	9.57 (3.27)	9.58 (3.48)	1.36	12.43	0.792 (0.585, 0.903)	0.12
M_DUR_ (ms)	10.07 (2.31)	10.72 (2.88)	1.0	6.61	0.877 (0.741, 0.944)	0.01
*Rectus femoris*						
RMS EMG (mV)	0.18 (0.82)	0.18 (0.81)	0.02	13.76	0.852 (0.698, 0.931)	< 0.00
RMS.M_MAX_ (%)	6.16 (1.97)	6.16 (1.76)	0.96	13.89	0.721 (0.38, 0.89)	0.14
H_MAX_.M_MAX_ (%)	18.80 (9.94)	15.65 (7.23)	5.66	16.73	0.644 (0.151, 0.887)	< 0.00
M_MAX_ (mV)	2.97 (0.75)	2.73 (1.09)	0.47	15.04	0.561 (0.139, 0.813)	< 0.00
M_DUR_ (ms)	9.95 (2.42)	10.02 (1.87)	1.0	6.01	0.899 (0.734, 0.963)	0.16
P_MAX_ (W)	179.7 (42.16)	183.33 (42.16)	9.80	4.03	0.945 (0.881, 0.975)	0.13
T0 (N.m)	119.78 (27.73)	115.84 (25.47)	6.55	4.82	0.938 (0.865, 0.972)	0.15
T_OPT_ (N.m)	48.40 (9.13)	47.83 (8.52)	2.43	3.75	0.928 (0.843, 0.967)	0.1
V0 (rad.s^−1^)	8.50 (1.88)	8.63 (1.92)	0.62	5.97	0.897 (0.781, 0.953)	< 0.00
V_OPT_ (rad.s^−1^)	3.42 (0.46)	3.54 (0.48)	0.17	4.13	0.852 (0.692, 0.932)	0.05

*TE* typical error, *CV* coefficient of variation, *ICC* intra-class correlation coefficient (2,1 model). * *P* < 0.05

### Voluntary EMG, M-wave and H-reflex

**Fig. 3:**
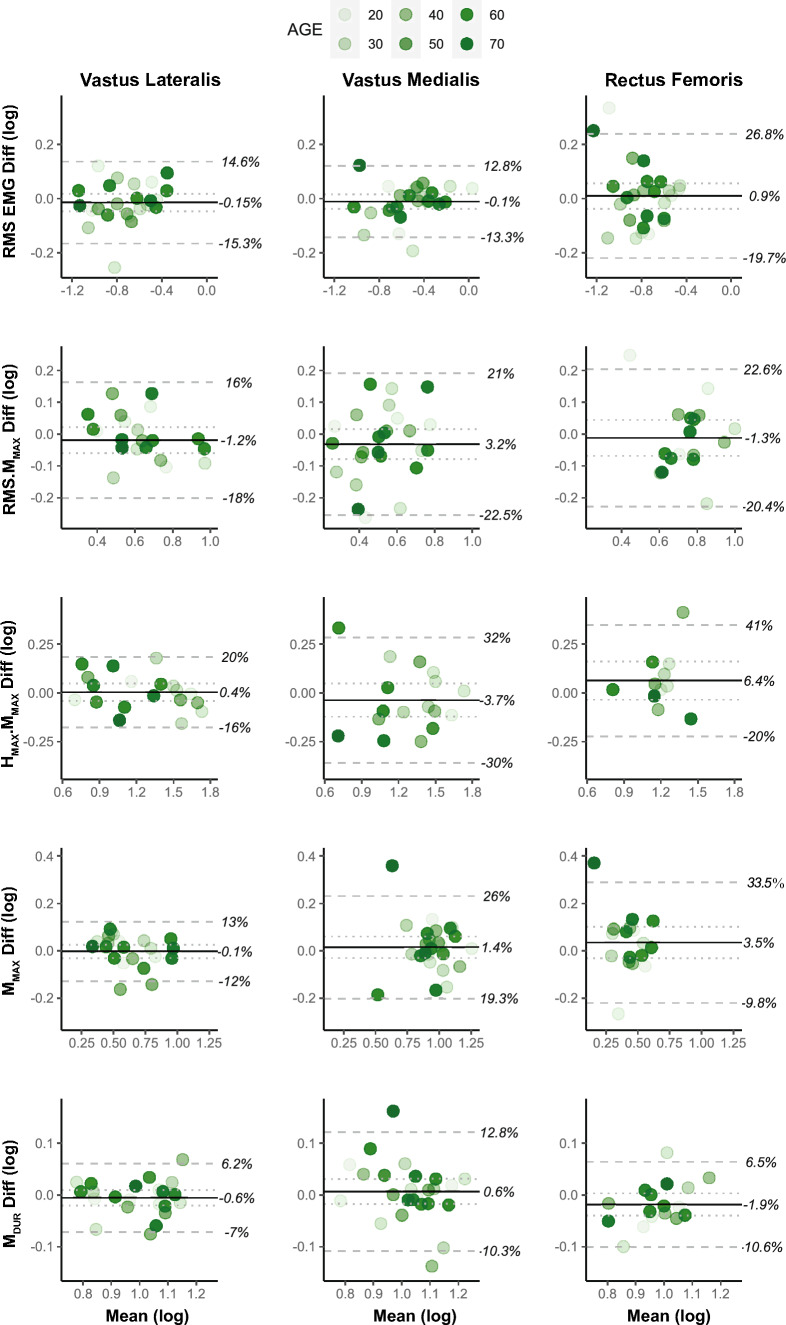
Bland–Altman plots with 95% level of agreement for *vastus lateralis* (left column), *vastus medialis* (centre column) and *rectus femoris* (right column) muscles. The mean value for sessions one and two is plotted on the x-axis, whereas the difference between sessions one and two is plotted on the y-axis. The solid line represents the grand mean difference for all participants, and the dotted line shows the 95% confidence interval. The dashed lines represent the lower and upper limits of agreement as a % difference (antilog of log-transformed data). Shading of the individual measures increases as a function of age. Sample size (n) for each variable: VL RMS EMG (23), VM RMS EMG (26), RF RMS EMG (26), VL RMS.M_MAX_ (21), VM RMS.M_MAX_ (25), RF RMS.M_MAX_ (17), VL H_MAX_.M_MAX_ (19), VM H_MAX_.M_MAX_ (17), RF H_MAX_.M_MAX_ (11), VL M_MAX_ (21), VM M_MAX_ (25), RF M_MAX_ (17), VL M_DUR_ (21), VM M_DUR_ (25), RF M_DUR_ (17)

Original data from 8 of the 15 EMG variables displayed a non-normal distribution. Consequently, all EMG variables were log transformed prior to analysis to permit investigation into inter-muscular differences in the outcomes and to permit meaningful interpretation of the LOA (Balshaw et al. 2017). EMG, M-wave and H-reflex responses were similar for sessions one and two for all quadriceps muscles (all *P* > 0.05), and the difference in the outcomes between sessions was not significantly different between the muscles (i.e. no interaction effects; all *P* > 0.05). ICCs for all outcomes measured in the *vastus lateralis* muscle were > 0.8 (Table 1). RMS EMG, H_MAX_.M_MAX_ and M_DUR_ measured in the *vastus medialis* and RMS EMG and M_DUR_ measured in the *rectus femoris* also showed ICCs > 0.8. CoVs were lower in the *vastus lateralis* compared to the *vastus medialis* and *rectus femoris* muscles for 4/5 EMG outcomes, with all *vastus lateralis* outcomes showing a CoV of < 12%. The *vastus lateralis* showed no notable systematic bias (all <  ~ 1%) and less random error compared to the *vastus medialis* (with exception of RMS EMG) and *rectus femoris* muscles for all outcomes (Fig. 3). Feasibility for M-wave/H-reflex responses was 81/73% for *vastus lateralis*, 96/65% for *vastus medialis* and 65/42% for *rectus femoris*.

### Torque–velocity and power–velocity relationships

**Fig. 4:**
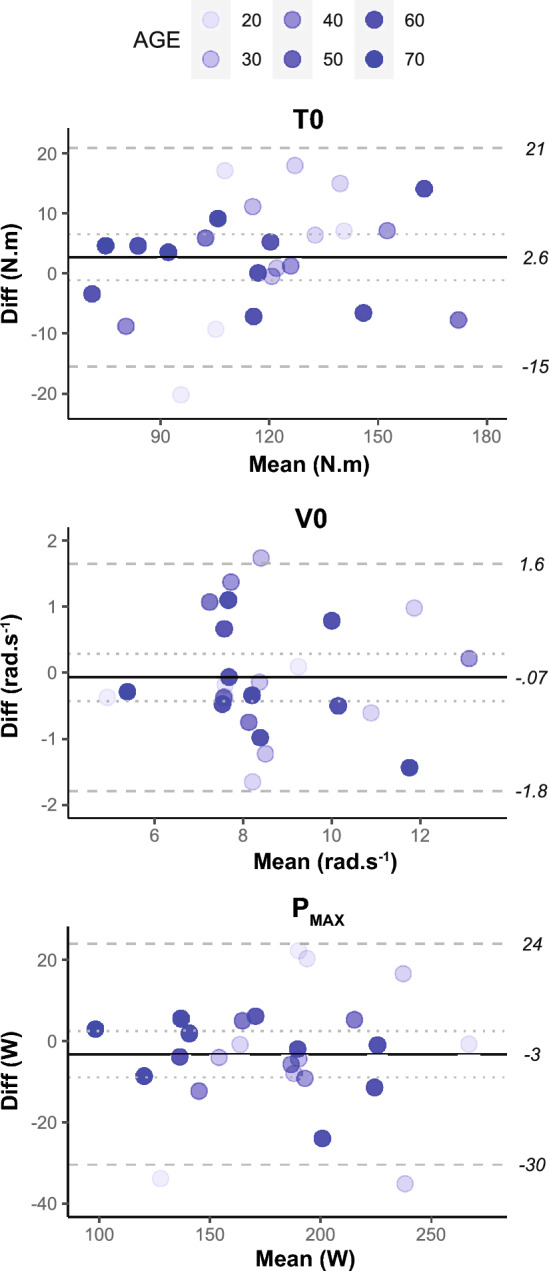
Bland–Altman plots with 95% level of agreement for variables extracted from the torque–velocity and power–velocity relationships. The mean value for sessions one and two is plotted on the x-axis, whereas the difference between sessions one and two is plotted on the y-axis. The solid line represents the grand mean difference for all participants, and the dotted line shows the 95% confidence interval. The dashed lines represent the lower and upper limits of agreement in original unit of measurement. Shading of the individual measures increases as a function of age. Sample size (n) for each variable: T0 (25), V0 (25), P_MAX_ (25)

All outcomes extracted from the torque–velocity and power–velocity relationships were similar between sessions (all *P* > 0.05). ICCs were > 0.8 for all outcomes (Table 1). CoVs for V0, T0 and P_MAX_ were ~ 6%, ~ 5% and ~ 4%, respectively. No notable systematic bias was identified for any of the outcomes (Fig. 4).

### Effect of age on reliability

Participant age was not associated with delta between sessions for 27 out of 28 outcomes (all *P* > 0.05) (Table 1), although increasing age increased the delta for VM H_MAX_.M_MAX_ (*R*^2^ = 0.42, *P* = 0.005).

## Discussion

All mechanical outcomes extracted from isometric maximal voluntary and evoked contractions demonstrated excellent relative and absolute reliability between sessions one and two. Similarly, all mechanical outcomes derived from the torque–velocity and power–velocity relationships constructed from maximal concentric contractions demonstrated excellent reliability between the sessions. The *vastus lateralis* muscle exhibited higher ICCs (all > 0.8) and lower CoVs (all < 12%) compared to the *vastus medialis* and *rectus femoris* muscles. Age was not correlated with the mean difference between sessions for 27/28 of the outcomes.

### Isometric voluntary and evoked forces

Quadriceps MVC, as a gold-standard measure of maximal muscle strength, demonstrated excellent inter-session reliability with an ICC of 0.869 and a CoV of 7.2%. Random error in strength measurements may be exacerbated in older adults due to large between-subject variability in neuromuscular performance (Hunter et al. 2016) or as they are generally less accustomed to high-force contractions compared to younger adults (Nuzzo et al. 2019). Our findings indicate that random error in MVC measurements may be dependent on overall voluntary strength capacity rather than age, as the mean difference in MVC between the sessions seemed higher when the mean MVC was lower, with no association with age (Fig. 2). Moreover, the overall mean difference at force levels <  ~ 150 N.m appeared in favour of higher values during the second session, indicating that females with lower strength capabilities may benefit from a second session to record maximal quadriceps isometric voluntary strength. Nonetheless, systematic bias was absent from MVC measurements in accordance with previous observations from isometric contractions (Nuzzo et al. 2019). Our method of measuring quadriceps MVC prior to impending supramaximal stimulation of the peripheral nerve did not appear to increase the random error in the measurement, as reliability was similar to previous findings from older females and when stimulation of the nerve was not applied (Holsgaard Larsen et al. 2007; Symons et al. 2004). Thus, the adopted method is advantageous in providing an elaborate evaluation of neuromuscular function without compromising MVC reliability. The reported LOA is acceptable for quantifying age-related differences in quadriceps MVC (Miller et al. 2021).

ICCs for quadriceps VA were not calculated due to the ceiling effect of the measurement (Place et al. 2007); however, the CoV was 2.6%, and the upper and lower LOA were 7.5% and − 9.8%, respectively (LOA ratio = 9.8%). Systematic bias was small, with a mean difference of 1.1% towards higher VA measurements during the second session (Nuzzo et al. 2019). These results represent slightly higher inter-session reliability and less random error in quadriceps VA measurements compared to younger males and females (Place et al. 2007; Behrens et al. 2017; Zech et al. 2008) and older patients with knee osteoarthritis (Staehli et al. 2010). However, many methodological factors can influence VA measurements (Nuzzo et al. 2019), which makes a physiological explanation for the small discrepancy in reliability difficult. The reported LOA must be considered when evaluating age-related deficits in voluntary activation of the quadriceps, as significant reductions of little as ~ 5% (Mau-Moeller et al. 2013) up to ~ 11% (Billot et al. 2014) have previously been reported.

All responses elicited from single- and double-pulse (100 Hz and 10 Hz) stimulations exhibited excellent reliability, evidenced by ICCs ranging from ~ 0.85 to 0.91 and CoVs of <  ~ 5%. For the peak force responses, there was no evidence of systematic bias (≤ 0.6 N.m) and random error was <  ~ 11 N.m. These results are either similar or more reliable than those of previous findings obtained from younger males and females (Behrens et al. 2017; Place et al. 2007) and comparable to older adults with knee osteoarthritis (Staehli et al. 2010). Age may reduce random error in twitch responses as older adults have less muscle potentiation following a pre-conditioning contraction (Baudry et al. 2005), which is dependent on type II fibres that preferentially atrophy into older age (Hunter et al. 2016). Although single-pulse twitches were anecdotally more tolerable than doublets, single-pulse twitch force was ~ 2/3rds the magnitude of the doublet peak force and thus did not represent the true potential of intrinsic muscle contractility. High- and low-frequency twitch forces are also more valuable than single-pulse twitch force responses as age-related deficits in factors such as neuromuscular propagation (e.g. at the neuromuscular junction and along the sarcolemma; 100 Hz) and excitation–contraction coupling (e.g. myofibrillar calcium kinetics and sensitivity; 10 HZ) can be evaluated (Millet et al. 2011; Hunter et al. 2016). It is also worth noting that no participants withdrew their voluntary consent to participate in the electrical stimulation protocol and all returned for the second retest session. Thus, the stimulating procedure was feasible in evaluating peripheral contractile skeletal muscle characteristics in healthy younger and older females. However, for more frail elderly or clinical groups, alternate (albeit limited) methods may be more appropriate (e.g. reducing supramaximal intensity) (Millet et al. 2011; Staehli et al. 2010).

### Electromyography

All quadriceps muscles showed excellent reliability for RMS EMG, though *vastus lateralis* was the only muscle to show excellent reliability for RMS.M_MAX_ (ICC = 0.85). M-wave responses and H_MAX_.M_MAX_ also showed excellent reliability in the *vastus lateralis* (ICCs > 0.95) and CoVs were lower compared to the other superficial quadriceps (Fig. 3). These outcomes were more reliable than reported previously for younger males (Doguet and Jubeau 2014; Jenkins et al. 2014; Balshaw et al. 2017; Hopkins and Wagie 2003) and M_MAX_ was comparable to older males and females with knee osteoarthritis (Staehli et al. 2010). The 5% MVC background force level adopted during H-reflex procedures may have improved reliability relative to previous studies, as females recruit more stable low-threshold motor units at higher firing rates to generate submaximal force levels compared to males (Guo et al. 2022). Thus, it may be more reliable to elicit quadriceps H-reflex responses at low background force levels when assessing females. However, similar to previous studies (Doguet and Jubeau 2014; Hopkins and Wagie 2003; Mau-Moeller et al. 2013), H-reflexes were not always identifiable for all quadriceps muscles and participants, potentially due to short durations between M-wave and H-reflex responses, depth of the femoral nerve in the femoral triangle or complex femoral nerve structure (e.g. branching, efferent to afferent fibre size ratios) (Hopkins and Wagie 2003). The LOA is acceptable for detecting age-related changes in RMS EMG and M-wave for both *vastii* muscles but may not be sensitive enough for *vastus medialis* H-reflex amplitude (Mau-Moeller et al. 2013). Lower reliability of VM H_MAX_.M_MAX_ in older adults (Table 1) also needs to be considered by future research, although the reasons for this observation are unclear. Age-related effects on *vastus lateralis* H-reflex amplitude have not previously been reported; however, H-reflex amplitude for the soleus ankle plantar-flexor muscle decreases as a function of age with deficits of ~ 20% reported in older adults (Baudry 2016). It is important to note that direct comparison on the reliability of voluntary and evoked EMG outcomes between studies is troublesome, as reliability is influenced by numerous factors including muscle length (Balshaw et al. 2017; Theodosiadou et al. 2023) and electrode location (Balshaw et al. 2017). Thus, the reliability of voluntary and evoked EMG responses should be established for specific protocols prior to experimentation.

*Vastus lateralis* EMG appeared more reliable compared to *vastus medialis* and *rectus femoris* muscles. ICCs were higher, CoVs were lower and the random error was smaller in the *vastus lateralis* for four out of five of the outcomes (Table 1 and Fig. 3), despite not reaching statistical significance. *Vastus lateralis* outcomes were also more reliable than findings reported for younger healthy males (Place et al. 2007; Doguet and Jubeau 2014; Jenkins et al. 2014; Balshaw et al. 2017). Of all the superficial quadriceps, *vastus lateralis* possesses a higher cross-sectional area (irrespective of age) (Mizuno et al. 2021), is the greatest contributor to the knee extensor moment (Zhang et al. 2003) and is the most reliable predictor of force output during knee extension and leg extension movements (Alkner et al. 2000). Thus, when evaluating age-related differences in female neuromuscular function, voluntary and evoked EMG outcomes may be more reliable in *vastus lateralis*. However, differences in *vastus lateralis* and *vastus medialis* absolute and normalized surface EMG responses do not adhere with similarities in the change in motor unit discharge rate observed during ramp contractions (i.e. common neural drive between the *vastii*) (Martinez-Valdes et al. 2018), suggesting that anatomical factors (e.g. muscle architecture, subcutaneous tissue thickness and cross-sectional area) or crosstalk (Farina et al. 2004) influences compound surface EMG responses measured from *vastus lateralis* and *vastus medialis* muscles.

### Torque–velocity and power–velocity relationships

All quadriceps torque–velocity and power–velocity characteristics exhibited excellent inter-session reliability (ICCs > 0.8) (Fig. 4). Inter-session reliability of quadriceps T0 between the two sessions was similar to measurements of quadriceps 1RM (do Nascimento et al. 2013) (although in that study, significant differences between the two sessions were observed) and isokinetic torques generated at 60°/s and 180°/s (Pincivero et al. 1997) in young and elderly males and females. Moreover, V0 and P_MAX_ showed lower CoVs than when measured separately from single isoinertial loads corresponding to 20%, 25% or 50% MVC in older males and females (Van Driessche et al. 2018; Bui et al. 2021). Our results show that measures of quadriceps maximal torque, velocity and power obtained from a torque–velocity test may provide more reliable and elaborate information regarding neuromuscular function compared to 1RM or isokinetic tests. Indeed, functional capacity in older adults is more strongly related to maximal muscular power than muscle strength (Byrne et al. 2016), and shortening velocity is a primary determinant of muscular power (Samozino et al. 2012; Byrne et al. 2016).

### Considerations

Although it is unclear whether a third experimental session could further improve the reliability of the outcomes, previous studies in younger males have shown negligible changes in maximal voluntary and evoked forces (Buckthorpe et al. 2012; Jenkins et al. 2014; Zech et al. 2008) and torque–velocity outcomes (Meylan et al. 2015). Thus, inconveniences imposed on participants (e.g. time commitment and exposure to peripheral nerve stimulation techniques) can be minimized, which increases the feasibility of implementing these techniques into future research protocols.

It is also worth noting that the average duration between experimental sessions of 9 ± 7 days may have in some cases led to eumenorrheic females (*n* = 7) being assessed at different phases of the menstrual cycle. Menstrual cycle phase was not controlled for as this was not expected to influence the mechanical outcomes (Nuzzo et al. 2019). The menstrual cycle phase may however influence motor unit discharge rate (Tenan et al. 2013) and be a source of random error in compound EMG outcomes, although sub-cohort comparisons between eumenorrheic participants and participants using contraceptives found no significant differences in reliability for any of the outcomes, and more research is needed using advanced neurophysiological techniques (e.g. high-density EMG) (Lulic-Kuryllo and Inglis 2022). Testing all eumenorrheic females in the same phase of their menstrual cycle would however have increased the test–retest duration to the duration of one menstrual cycle (i.e. ~ 28 days), thus introducing further and potentially larger sources of variability.

## Conclusions

This is the first study to provide a comprehensive analysis of the test–retest reliability of quadriceps maximal voluntary and evoked forces and electromyography outcomes used to evaluate the ageing neuromuscular system and adaptation to exercise, with special reference to females. The results demonstrate that reliable and accurate measurements can be obtained in one experimental session with no effect of age. This is important for future research aiming to quantify age-related degeneration in neuromuscular function and/or neuromuscular adaptation to exercise at different stages of the female lifespan.

## Data Availability

The data that support the findings of this study are available from the corresponding author upon reasonable request.
